# Epidemiologic Features of Kawasaki Disease in Japan: Results from the Nationwide Survey in 2005-2006

**DOI:** 10.2188/jea.JE2008001

**Published:** 2008-08-07

**Authors:** Yosikazu Nakamura, Mayumi Yashiro, Ritei Uehara, Izumi Oki, Makoto Watanabe, Hiroshi Yanagawa

**Affiliations:** 1 Department of Public Health, Jichi Medical University

**Keywords:** Mucocutaneous Lymph Node Syndrome, Incidence, Cardiovascular Diseases, Immunoglobulin, Intravenous, Epidemiology

## Abstract

**Background:**

The most recent epidemiologic features of Kawasaki disease are unknown.

**Methods:**

The 19th nationwide survey of the disease was conducted in 2007, targeting patients who were affected by this disease in 2005 and 2006. All pediatric departments in hospitals with 100 or more beds and pediatric hospitals were asked to report all Kawasaki disease patients during the 2 survey years.

**Results:**

From 1543 departments and hospitals, a total of 20475 patients (10041 in 2005 and 10434 in 2006) were reported. There were 11892 male patients and 8583 female patients. The average annual incidence rate was 184.6 per 100000 children aged 0-4 years. The number of patients and the incidence rate have increased significantly during the past 12 years. The age-specific incidence rate was distributed monomodally with a peak at 6-8 months of age. The prevalence of cardiac lesions in the acute phase of the disease and of cardiac sequelae were higher among infants and old patients.

**Conclusion:**

The number of patients with Kawasaki disease and its incidence rate in Japan are continuously increasing.

## INTRODUCTION

Kawasaki disease is a syndrome of unknown etiology. It usually affects infants and toddlers and causes systemic vasculitis.^1^^,^^2^ Cardiac sequelae, such as coronary artery aneurysms, are one of the most important aspects of this disease.^2^^-^^6^

Since 1970, nationwide epidemiologic surveys of Kawasaki disease have been conducted almost every 2 years, and several epidemiologic features of the disease in Japan have been revealed through these surveys.^7^^-^^9^ This paper presents the results of the 19th survey, which was conducted in 2007 and targeted patients who were affected in 2005 and 2006. This paper focuses on the main epidemiologic features. We will discuss the clinical features in detail in other articles in the near future.

## METHODS

This is a retrospective incidence survey that included patients with Kawasaki disease who visited the target hospitals for the first time during the 2-year period from January 2005 through December 2006.

The following medical facilities were requested to participate in the survey: (1) all hospitals with a pediatric department and a bed capacity of 100 or more or (2) specialized pediatric centers, even if their bed capacity was less than 100. The retrospective incidence survey method has been used since the first nationwide survey in 1970.^10^ In January 2007, questionnaire sheets and diagnostic guidelines created by the Japan Kawasaki Disease Research Committee^11^ were mailed to those in charge of the pediatric department of their respective hospitals. The prepared list of hospitals for the survey was based on the “Listing of Hospitals 2003-2004” compiled by the Committee on Studies of Health Policies, Ministry of Health, Labour and Welfare. There were 2223 facilities that met the conditions stated above.

The questionnaire form contained questions regarding the following: address (municipality), sex, date of birth, date and day of illness at first hospital visit, days of illness when discharged from the hospital, day of illness when fever declined, diagnosis, lesion at the site of bacille Calmette-Guérin (BCG) inoculation, immunoglobulin therapy, recurrences, patient’s siblings, parents’ prior history of Kawasaki disease, and cardiac lesions. Cardiac lesions were classified as acute conditions that developed within 1 month of onset (acute lesions) and those that persisted for 1 month after onset (sequelae). Almost all patients were diagnosed using two-dimensional echocardiography.

After checking for possible inconsistencies, the form was sent back to the respondents to correct any errors. The incidence rates were based on the population data reported in the Vital Statistics of Japan.^12^

To evaluate the yearly increase in the number of patients and the incidence rate, several regression models were assumed using data collected over 12 years-from 1995, when the recent increase in the number of patients and incidence rate started, through 2006. Because the response rates in the surveys were not 100%, data corrected for the response rates (crude data divided by response rates) as well as crude data were analyzed. The Statistical Package for the Social Sciences (SPSS)^®^ ver. 15.0J was used for the analyses.

The Ethical Board of Jichi Medical University approved this survey (July 18, 2006, No. 06-08).

## RESULTS

Of the 2223 invitations to the survey, 40 were returned because the relevant pediatric department or the institution itself had closed down. Of the remaining 2183 departments, 1543 (70.7%) responded to the survey. From these departments, a total of 20475 patients (10041 in 2005 and 10434 in 2006) were reported. There were 11892 male patients and 8583 female patients. The average annual incidence rate for the observed 2 years was 184.6 per 100000 children aged 0-4 years (209.3 in the case of males and 158.6 in the case of females).

**Figure 1.**
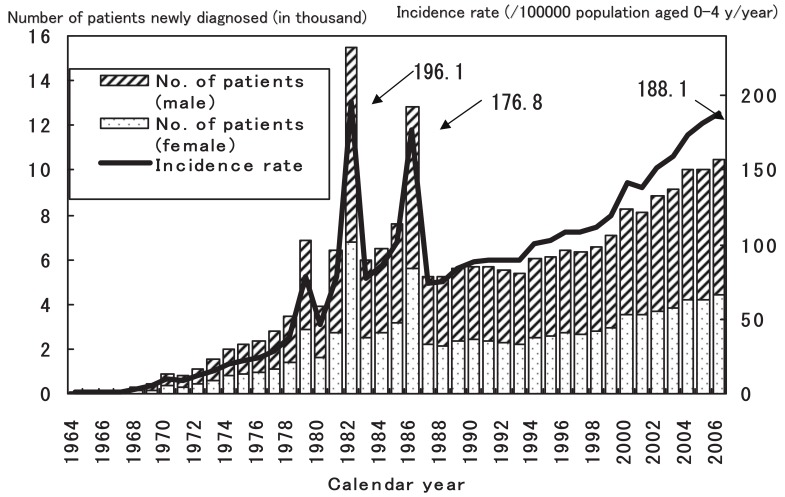
The number of patients with Kawasaki disease and incidence rate in Japan by calendar year.

The annual numbers of patients with Kawasaki disease and the incidence rates of this disease in the past 19 nationwide surveys, including the current one, are shown in Figure 1. As previously reported, there were 3 nationwide epidemics of this disease in Japan–in 1979, 1982, and 1986. Since then, no nationwide epidemic was observed, but the number of patients started to increase in the mid 1990s. Because of the decreasing birth rate in Japan, the incidence rate has increased more rapidly than the number of patients, reaching 188.1 per 100000 children aged 0-4 years in 2006. This figure is higher than those in 1979 and 1986 when the nationwide epidemics occurred.

To mathematically evaluate the increase in the number of patients and the incidence rates, linear models, quadratic models, cubic models, logarithmic models, reciprocal models, and second power models were used as regression models. Of all the models, linear regression models were the best fitted, and the results are shown in Table 1. All coefficients of determination were greater than 0.95, indicating that both the number of patients and the incidence rate have increased linearly during the past 12 years.

**Table 1.** Results of linear regression analyses of the annual number of patients and the incidence rate of Kawasaki disease in Japan.

			Coefficient for calendar year^*^	Constant	Coefficient of determination	*P*
No. of patients	Without correction for response rate	Males	251.2	-497861.9	0.963	< 0.001
		Females	180.5	-357665.8	0.963	< 0.001
		Both	431.7	-855527.8	0.996	< 0.001

	With correction for response rate	Males	333.3	-659854.8	0.949	< 0.001
		Females	239.2	-473561.9	0.946	< 0.001
		Both	572.5	-1133416.7	0.951	< 0.001

Incidence rate**	Without correction for response rate	Males	9.5	-18902.2	0.962	< 0.001
		Females	7.2	-14310.0	0.967	< 0.001
		Both	8.4	-16663.0	0.966	< 0.001

	With correction for response rate	Males	12.8	-25285.3	0.958	< 0.001
		Females	9.7	-19129.4	0.960	< 0.001
		Both	11.2	-22283.8	0.961	< 0.001

* : 1995-2006

** : per 1 million population aged 0-4 years per year

**Figure 2.**
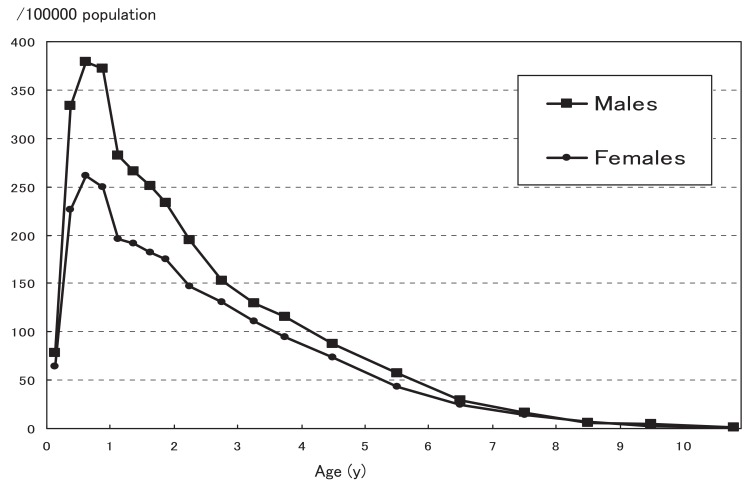
Age-specific annual incidence rate of Kawasaki disease in Japan in 2005-2006.

Age-specific incidence rates for each sex are shown in Figure 2. Consistent with previous data, the incidence rate was the highest in the latter half of the first year of life, and gradually decreased with age. In the 18th survey,^9^ the highest incidence rate was observed among those aged 9-11 months, whereas in the current survey, it was observed among those aged 6-8 months.

Among the 20475 cases reported to the survey, there were 16818 (82.1%) typical definite cases, i.e., those with 5 or 6 of the symptoms mentioned in the diagnostic guidelines for the disease; 722 (3.5%) atypical cases, i.e., those with 4 of the 6 diagnostic symptoms and coronary aneurysms, including dilatation; and 2935 (14.3%) suspected cases. In the suspected cases, the diagnostic criteria were not satisfied, but Kawasaki disease was suspected by the treating pediatricians.

A total of 259 (1.3%) patients had 1 or more siblings affected by Kawasaki disease, and 102 had 1 or both parents with a history of the disease. Further, 755 (3.7%) had recurrent disease, and 2 fatalities were reported.

**Figure 3.**
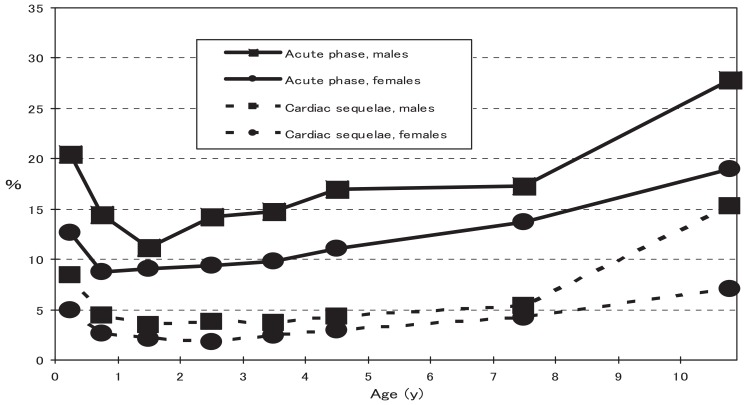
Age-specific prevalence of cardiac lesions and sequelae due to Kawasaki disease in Japan in 2005-2006.

In the acute phase, 2650 (12.9%) patients showed the following cardiac findings: giant coronary aneurysms (71 patients, 0.35%), coronary aneurysms (305 patients, 1.49%), coronary dilatations (2,065 patients, 10.1%), coronary stenoses (5 patients, 0.02%), myocardial infarctions (3 patients, 0.01%), and valvular lesions, (385 patients, 1.9%). At 1 month after disease onset, the following cardiac sequelae were reported in 780 (3.8%) patients: giant coronary aneurysms (71 patients, 0.35%), coronary aneurysms (212 patients, 1.0%), coronary dilatations (477 patients, 2.3%), coronary stenoses (12 patients, 0.06%), myocardial infarctions (3 patients, 0.01%), and valvular lesions (84 patients, 0.4%). As shown in Figure 3, the proportions of the cardiac findings were higher in males than in females, and among infants and old patients.

## DISCUSSION

In this paper, we present the most recent epidemiologic features of Kawasaki disease in Japan by using the results of the 19th nationwide survey. Nationwide surveys have been conducted almost every 2 years since 1970.^7^^-^^9^ As shown in Figure 1, the number of patients and the incidence rate dramatically increased during the recent decade. If the annual incidence rate, which was 188.1 per 100000 children aged 0-4 years in 2006, increases as shown by the linear regression model (Table 1) (incidence rate = 8.4 × calendar year -16663.0), that in 2007 will be 195.8, which is nearly equal to the highest annual rate reported–196.1 in 1982–and that in 2008 will be 204.2. Because the etiology of this disease remains unknown, the reason for this increase is also unknown. If this disease has an infectious etiology, changes in the toxicity of the causative microorganisms might be responsible for the increase in the incidence rate. Another possible reason is a change in the susceptibility of the hosts.

In this survey, a response rate of 70.7% was obtained after sending 2 reminders. Therefore, the actual number of patients must be higher than that observed. Further, by using some data,^13^ we estimated that the actual number of patients was not more than 10% greater than that shown in this article.

There are some potential factors that distort the observed chronological changes in the epidemiology of the disease. One of these is the response rate of the survey–70.7% in the current survey. The response rate was 70.1% in the 18th survey (2003-2004),^9^ 68.0% in the 17th survey (2001-2002),^8^ 66.5% in the 16th survey (1999-2000),^8^ 68.5% in the 15th survey (1997-1998),^14^ and 67.7% in the 14th survey (1995-1996).^14^ Thus, the response rates were similar, and we do not consider that changes in the rate affected the chronological changes. Further, differences in diagnostic criteria may also have influenced the results. In the current survey, the fifth revised version of the diagnostic guidelines for Kawasaki disease, which was issued in 2002, was used. Although the guidelines were revised based on the increase in the knowledge about the disease since the first nationwide survey in 1970, the principle points were not changed;^11^ therefore, the revision of the guidelines was not responsible for the chronological change. If the number of suspected cases increases, the clinical features of Kawasaki disease also change. The proportion of suspected cases was 14.3% in the current survey and 13.6% in the 18th survey.^9^ Although no data regarding suspected cases was obtained before the 18th survey, the proportion of suspected cases does not seem to have increased and thus was not the reason for the increase in the annual numbers of patients and incidence rates.

On the age-specific incidence rate curve shown in Figure 2, the peak was 3 months earlier than that in the 18th nationwide survey.^9^ However, this does not conclusively prove that the disease affects younger children because the findings in this survey may be due to chance. The monomodal incidence rate curve indicated a possible relationship between disease occurrence and an infectious agent.^15^ The low incidence rate immediately after birth might be due to maternally acquired passive immunity, and the decrease in the incidence after 1 year of age might be caused by herd immunity.

One of the major problems associated with Kawasaki disease is the cardiac sequelae. Fortunately, the proportion of patients with these sequelae is decreasing annually. The proportion of patients with cardiac sequelae was 7.0% in the 15th nationwide survey (1997-1998),^16^ 5.9% in the 16th survey (1999-2000),^8^ 5.0% in the 17th survey (2001-2002),^8^ and 4.4% in the 18th survey (2003-2004).^9^ The proportion was more than 10% in the early 1990s.^17^ This decreasing trend is due to the progress in the diagnosis of the disease and cardiac lesions, and their treatment, the core of which is formed by intravenous immunoglobulin therapy. However, Kawasaki disease is the leading cause of acquired heart diseases in childhood both in Japan^18^ and the United States,^19^ and treatment to prevent sequelae must be improved. The reason for the high proportion of sequelae among infants might be due to their immature circulation systems, which are affected by the vasculitis caused by Kawasaki disease.^20^ Moreover, the high proportion among old patients might be due to the difficulty in diagnosis because some of these patients show an atypical clinical course of the disease.^21^

There are some limitations to the current survey. Because the etiology of the disease is currently unknown, no specific diagnostic findings of the disease have been outlined. Therefore, all patients reported in the survey were diagnosed by pediatricians based on diagnostic guidelines. Another problem is that some patients might be reported from more than 1 hospital because they were referred to other hospitals due to the severity of the disease. In the 18th nationwide survey, 8.9% of the patients were referred from other hospitals, some of which were not the target hospitals for the survey because of small capacity of beds or no pediatric departments, and 4,9% were referred to other ones (unpublished data). Therefore, the proportion of double registration in the current study might be lower than the abovementioned values, and its effect on the final results was estimated to be small.

In conclusion, the number of patients and the incidence rate of Kawasaki disease in Japan have increased annually, and the problems of cardiac sequelae persist. Monitoring of disease occurrence must be continued.
